# A new putative carlavirus identified by metagenomic analysis in a wild weed in Angola

**DOI:** 10.1007/s00705-026-06678-2

**Published:** 2026-06-12

**Authors:** Serafina Serena AMOIA, Annalisa Giampetruzzi, Luisa Flora Antònio, Aderito Tomàs Pais da Cunha, Angelantonio Minafra

**Affiliations:** 1 Institute for Sustainable Plant Protection – CNR, Bari, Italy; 2 Centro Nacional de Investigação Cientifica, Luanda, Angola; 3 Instituto Superior Politécnico do Cuanza Sul, Sumbe, Angola

## Abstract

A metagenomic analysis was performed by high-throughput sequencing (HTS) to identify viruses infecting a wild weed collected in Seles (Angola), which exhibited clear yellowing symptoms. The analysis led to the discovery of a putatively novel carlavirus, tentatively named ‘Seles weed carlavirus’. The complete genome sequence, consisting of 8,597 nucleotides, poly-A tail excluded, exhibited the typical organization of members of the genus *Carlavirus*, including the replicase polyprotein (ORF1); the triple gene block (ORFs 2–4); the coat protein (ORF5) and an RNA-binding protein (ORF6). The replicase polyprotein and coat protein gene regions of the newly described virus shared the highest amino acid sequence identity with the corresponding sequences of cowpea mild mottle virus (51.40%) and Hainan betaflexivirus (63.08%), respectively. The infection was further confirmed by RT-PCR with multiple specific targeted primer pairs, whose related amplicons were cloned and sequenced.

**Supplementary Information:**

The online version contains supplementary material available at 10.1007/s00705-026-06678-2.

In developing countries, as in the Sub-Saharian tropical areas, there is the compelling need to contain virus diseases of crops to increase yield and ensure sustainable food supply [1]. In these regions, dangerous vector-borne plant pathogens can be easily spread in the absence of effective containment measures [2]. To address this issue, the application of sensitive monitoring technologies, such as high-throughput sequencing, to investigate metaviromes in agro-ecosystems is greatly improving the detection of known and previously unidentified viral and viroid pathogens [3, 4].

In the framework of the EU-funded RE-FARM project, a survey was conducted in June 2024 to assess virus-derived symptoms across experimental fields located in the provinces of Benguela and Cuanza Sul, Angola. Among the inspected crops, a few plants of a wild weed (referred as ‘*ndindje’* in vernacular language), growing close to intercropped maize and bean cultivated field in Sungo do Galo (Municipality of Seles), exhibited virus-like symptoms. Based on local knowledge, the plant had been tentatively identified as a member of the Solanaceae family. Symptoms included marked leaf chlorosis and distortion (Fig. 1), therefore representative leaf samples were collected for a virome study.

**Fig. 1:**
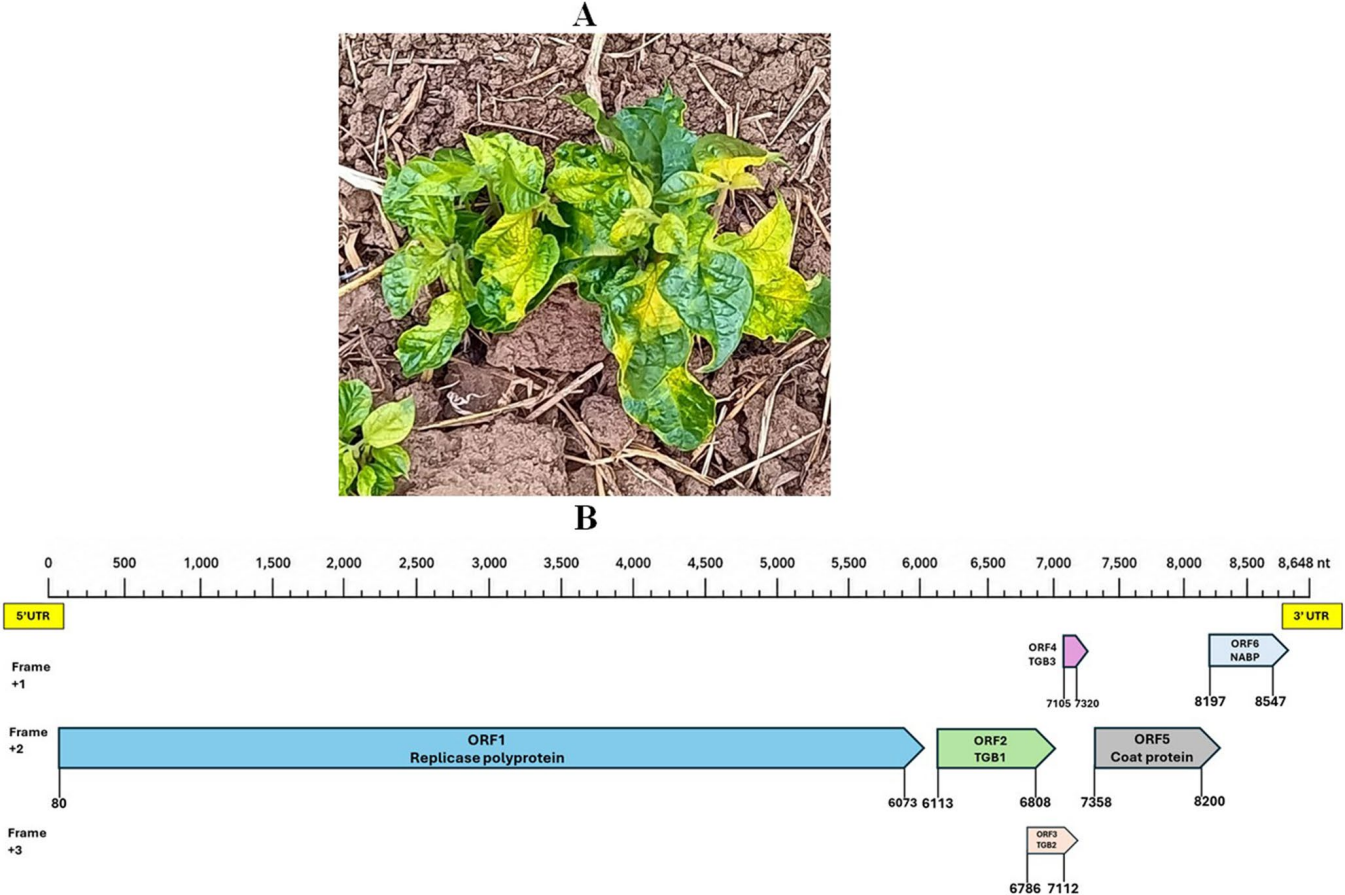
(**A**) Chlorotic symptoms observed on leaves of a wild weed during a field survey conducted in Cuanza Sul province of Angola (June 2024). (**B**) Schematic representation of the genome organization of Seles weed carlavirus. The predicted open reading frames (ORF1–ORF6) and coordinates are also indicated in the respective frames

Total RNA was extracted from leaf tissues using a CTAB method and LiCl precipitation [5]. Optical density measurement and gel electrophoresis inspection were evaluated for the sample before sending for high throughput sequencing (rRNA-depleted total RNA protocol) to an external service (Macrogen, South Korea). The sequencing was carried out on an Illumina NextSeq2000 platform (2 × 150 bp), obtaining 58,196,570 raw reads. The quality of the raw reads was assessed by the FastQC tool (https://www.bioinformatics.babraham.ac.uk/projects/fastqc/) and the adapter trimming was performed using BBDuk implemented on the Geneious Prime^®^ platform. *De novo* assembly was performed using SPAdes software v3.11.1 [6], either on Geneious Prime^®^ or Galaxy platform, retrieving a total of 13,857 contigs. Those contigs were annotated using BLASTN against the non-redundant nucleotide plant virus database (customly selected from https://www.ncbi.nlm.nih.gov/nucleotide/; accessed on 15 December 2025) for the identification of known viruses. Subsequently, BLASTX matching was applied against a similarly customized database of plant viral structural and functional proteins, searching for possible new viral species.

The thresholds were set at an e-value of < 10^− 4^, a percent identity higher than 50% on an alignment length of at least 50 amino acids. At the end of the process, 347 contigs of variable lengths (from a few hundred (ca. 300) to several thousand (8,500) nucleotides), matching with plant virus sequences, were positively selected.

Among the different contigs that could be traceable to or identified as plant virus-derived sequences, few of them were addressed because of their features of similarity with members of the *Carlavirus* genus [7]. Among those, one was selected, spanning a length of 8,551 nt excluding the polyA tail. Partial amino acid sequences from this selected contig (in particular the ORF1 product and the coat protein (CP)) were aligned and compared for pairwise identity using the Clustal Omega tool (EMBL-EBI; https://www.ebi.ac.uk/jdispatcher/msa/clustalo; [8]) with a panel of amino acid sequences from viruses that matched with the highest similarity according to BLASTP hits (Table 1). Amino acid sequences derived from ORF1 and ORF5 (coding for the CP), were subsequently used for phylogenetic analysis by the maximum-likelihood (ML) method, based on the LG (*+ F*) and cpREV (*+ F)* best-fit models, respectively, with a bootstrap test of 1,000 replicates, using MEGA X software v. 12.1.2 [9]. Two diagnostic primer sets were designed using multiple alignments of genomes and manually searching for regions showing the highest nt conservation between the viral sequence from this study and references (Suppl. Table 1). The primers targeted regions of 452 and 500 bp, within the CP and the replicase gene, respectively. Starting from 500 ng of total RNA extracts, random hexamers-primed cDNA was synthesized, using M-MLV RTase (Thermo Fisher Scientific), and used as template for standard PCR. PCR reaction mix was set up as follows: 2x GoTaq^®^ Green Master Mix (Promega), 200 nM of each primer, 1 µL of cDNA and nuclease-free H_2_O into a 20 µL final volume. The PCR program included: one cycle at 95°C for 3 min, 35 cycles at 94°C for 30 s, 58°C for 30 s, and 72°C for 30 s, followed by a final extension at 72°C for 10 min. The amplified fragments were cloned using the StrataClone pSC-a system (Stratagene, Agilent Technologies) and the obtained purified plasmids were Sanger-sequenced in both directions. The 5’ terminal sequence of the putative novel carlavirus genome was searched with 5’-rapid amplification of cDNA end (RACE) experiment using the 5’ RACE Kit, 2nd Generation (Roche^®^ Life Sciences), with the specific reverse primers *race2* for cDNA priming and *race1* for the subsequent PCR on the oligo-dA-extended cDNA (Suppl. Table 1), according to the manufacturer’s protocol. The resulting amplicon, showing the expected size of approximately 200 bp, was cloned as described above for sequence verification. Moreover, detailed alignment of the complete set of the reads in the library, mapping to the selected, near-full length genome contig, was inspected in Geneious Prime^®^ version 2020.0.5, at 5’ and 3’ untranslated regions (UTRs) as well as on the sites of both diagnostic amplifications within the ORF1 and ORF5, to further confirm primer matching. The consensus sequence among the reads alignment at the 5’ genome terminus matched with that derived from the amplified fragment and is shown in Suppl. Figure 1 A. On the other hand, all the 3’-coterminal reads shared the same sequence in the 3’ UTR upstream of the poly(A) tail (Suppl. Figure 1B).

**Table 1:** Pairwise identity of the Seles weed carlavirus genes with homologous counterparts in some selected carlaviruses

Virus	ORF1 (% nt/aa)	CP (% nt/aa)
Ligustrum virus A(NC_031089.1)	53.79/49.44	57.93 /48.89
jasmine virus C-isolate A31(NC_030926)	53.94/48.78	61.57/57.62
Papaya mottle-associated virus (NC_076712.1)	51.30/40.93	60.07/57.93
potato virus M(NC_001361)	53.34/46.67	56.88/53.05
pepper virus A(NC_034376)	57.34/47.77	61.59/58.33
cowpea mild mottle virus(NC_014730)	57.09/51.40	58.03/55.76
Hippeastrum latent virus(NC_011540)	52.86/47.58	59.97/53.24
carnation latent virus(NC_038865)	51.94/43.03	51.79/39.56
Hainan betaflexivirus(MW897313.1)	57.69/49.50	62.25/63.08
yam latent virus(NC_026248)	54.29/48.40	59.02/52.54

ORFinder (https://www.ncbi.nlm.nih.gov/orffinder/) and CD Search (https://www.ncbi.nlm.nih.gov/Structure/cdd/wrpsb.cgi) analyses of the putative full length genome sequence (8,597 nt) revealed the typical genome organization and conserved motifs of members of the *Carlavirus* genus.

The genome organization includes a 79 nt-long 5’ UTR. The ORF1 (nt 80 − 6,073; 1,997 aa for a predicted polyprotein product of 224.8 kDa) contains the domains of the viral methyltransferase (aa positions 44–354), AlkB (aa 707–840), carlavirus C23 peptidase (aa 1,004 − 1,090), viral helicase1 (aa 1,187-1,451) and finally, the RNA-dependent RNA polymerase (RdRp) domain (aa 1645–1962). An intergenic region of 40 nt separates ORF1 from the triple gene block genes (TGB; ORF2, 3 and 4). ORF2, encoding the TGB1, spans positions 6,113 to 6,808 (696 nt/231aa; 25.7 kDa); ORF3 (TGB2) extends from 6,786 to 7,112, is 327 nt-long (108 aa; 11.5 kDa), whereas ORF4 (TGB3; positions 7,105 to 7,320) is 216 nt-long (71aa; 7.9 kDa). After a second short intergenic region of 38 nt, ORF5, corresponding to the CP gene, is located, being 843 nt/280 aa long for a 31.2 kDa protein. The final ORF6 (350nt/116aa) codes for a 13 kDa protein, containing a C4-type zinc finger domain with a putative function of RNA binding and viral transcription regulation.The 3’ non-coding region spans 50 nt upstream of the poly(A) tail. No 3’-RACE was performed on the viral RNA, since a unique consensus sequence before the poly(A) tail was reconstructed from co-terminal reads (Suppl. Figure 1B).

The carlavirus-typical motif C/TTTAGGT is located in the short intergenic regions upstream of both the triple gene block and the coat protein gene, and it could play a role as ribosome binding site for translation of sub-genomic viral mRNAs [10].

The ORF1 and the CP amino acid sequences of this putative carlavirus were used to reconstruct the phylogeny of a selected array of viruses within the genus. In both the ML phylogenetic trees (Fig. 2, A and B), the virus clustered with the same references (i.e., papaya mottle-associated virus (PapMaV), pepper virus A (PepVA), Hainan betaflexivirus and cowpea mild mottle virus (CPMMV)) and it has, as proximal nodes, the Hippeastrum latent virus (HLV)and jasmine virus C (JVC).

**Fig. 2:**
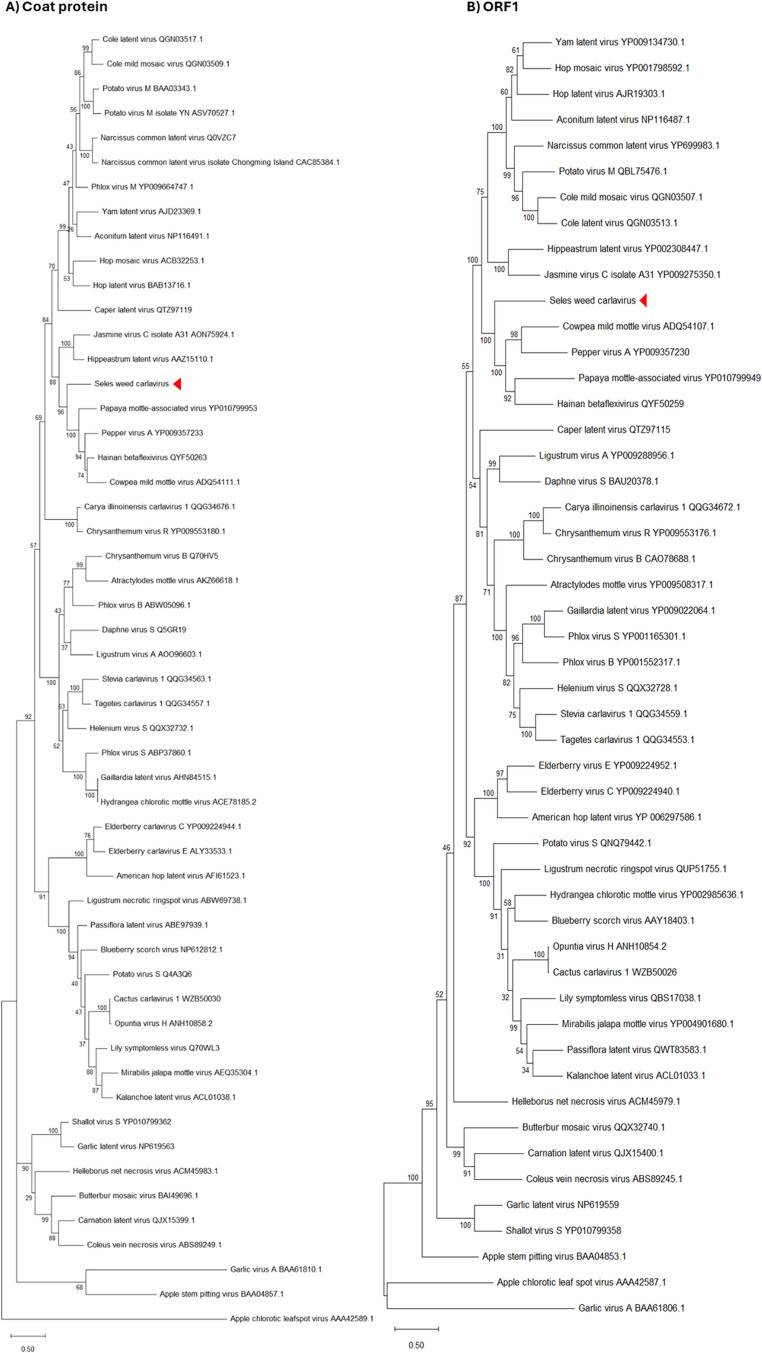
Phylogenetic trees constructed using the alignments of amino acid sequences of (**A**) the coat protein and (**B**) the ORF1 polyprotein of representative members of the genus *Carlavirus*. The trees were generated using the maximum-likelihood method (cpREV with Freqs. (+ F) and LG with Freqs. (+ F) models, respectively). Branch support was evaluated by bootstrap analysis with 1000 replicates. Scale bars located below trees indicate the number of amino acid substitutions per site. A red triangle indicates the newly Seles weed carlavirus described in this study. Three additional reference virus sequences, belonging to species in *Betaflexiviridae* have been included as outgroup (garlic virus A, apple stem pitting virus and apple chlorotic leafspot virus)

Along the whole ORF1 polyprotein, pairwise amino acid identity ranged from 40.93% ( PapMaV) to 51.40% (CPMMV), while the CP shows the highest identity with the cognate gene of Hainan betaflexivirus with 63.08% (Table 1). This latter virus sequence was also described in a metagenomic study, not even regarding an infected plant [11]. These identity values were lower than those established by ICTV as species demarcation criteria for the betaflexiviruses [7]. Therefore, we could consider this genomic sequence as belonging to a putatively novel virus in the *Carlavirus* genus, that has been provisionally named ‘Seles weed carlavirus’. The genome sequence of this putative new member in the family *Betaflexiviridae* has been deposited in GenBank under acc.nr. PX870806.

The virus sequence was confirmed by Sanger sequencing of the cloned amplicons derived from the same total RNA extract submitted for HTS. The amplified fragments matched the corresponding contig sequence with 99% nt identity for both amplicons, indicating that a low degree of variability was present in the genomic RNA. This analysis was consistent with the reads alignment (Suppl. Figure 1, A and B).

An analysis of putative recombination events, performed using RDP4 software version 4.39 [12], indicated that no significantly supported recombination events, involving Seles weed carlavirus and the selected subset of carlavirus genomes included in the phylogenetic analysis, had occurred .

A preliminary BLASTN analysis of the transcript RNAs from the host plant suggested that it could belong to the Lamiaceae family (data not shown), different from the previous taxonomic attribution gathered by local farmers.

Carlavirus reads accounted for 0.024% of the total reads in the library. However, the possible presence of another virus, co-existing with the Seles weed carlavirus, was revealed by a number of reads (around 0.005% of the total), mapping to several begomovirus sequences (data not shown). Therefore, the striking discoloration observed on the affected plants cannot be specifically associated with the newly characterized virus, since at least two viruses were simultaneously revealed in the sample. The occurrence of mixed viral infections in weed hosts is epidemiologically relevant because these plants may act as reservoirs facilitating virus persistence and vector-mediated transmission, leading to possible emergence of viral diseases in neighboring crops. Indeed, the Seles weed carlavirus belongs to a genus whose members are known to be efficiently transmitted mainly by aphids and quite widespread on several crops. Spill-over events could potentially occur [13] and contribute to the emergence and spread of new diseases within the same agro-ecosystem and on different, potentially susceptible, crops. The present study highlights that the integration of metagenomic investigation with early-warning systems for the potential spread of virus pathogens should be complemented by systematic visual inspections and diagnostic monitoring within agro-ecosystems. This combined approach is essential for protecting food supply chains and for limiting viral disease outbreaks.

## Supplementary information

Below is the link to the electronic supplementary material.

Supplementary Material 1 (DOCX 626 KB)

## Data Availability

This metagenome project has been deposited in the GenBank repository. The complete genome sequence of Seles weed carlavirus has been deposited under the accession number PX870806 (BioProject: PRJNA1399677; BioSample: SAMN54503719-Ang_D (TaxID: 4107; SRA: SRR36723783).

## Declarations

### Disclaimer

This publication has been produced within the framework of the RE-FARM project (Research on agroecological innovations for increasing resilience to climate change in Cuanza Sul and Benguela), financed by the European Union under the DeSIRA initiative (FOOD/2021/429-168) and implemented by Università degli Studi di Firenze (UNIFI), in partnership with COSPE and Centro Nacional de Investigação Científica (CNIC).
