# Supplementary material for: A new putative carlavirus identified by metagenomic analysis in a wild weed in Angola
Source: Arch Virol. 2026 Jun 12;171(7):207. doi: 10.1007/s00705-026-06678-2 (PMC13260034; doi:10.1007/s00705-026-06678-2)
Supplement: Supplementary file 1 — Supplementary Material 1 (DOCX 626 KB) [file 705_2026_6678_MOESM1_ESM.docx]

**Supplementary files**

**A new putative carlavirus identified by metagenomic analysis**

**in a wild weed in Angola**

Amoia Serafina Serena^1^, Giampetruzzi Annalisa^1^, António Luisa Flora^3^, Pais da Cunha Adérito Tomás^2,3^, Minafra Angelantonio^1#^

^1^ Institute for Sustainable Plant Protection – CNR, Bari, Italy

^2^ Centro Nacional de Investigação Cientifica, Luanda, Angola

^3^ Instituto Superior Politécnico do Cuanza Sul, Sumbe, Angola

^#^Corresponding author: angelantonio.minafra@cnr.it

| **Supplementary Table 1.** List of primers used in the study. | | |
| --- | --- | --- |
| **Primer name** | **Sequence 5’- 3’** | **Amplicon size (nt)** |
| CP_For | GAGGCCCGTACTACACATGG | 452 |
| CP_Rev | AGTCAAATGCAGCCATCCGA |  |
| Rep_for1 | CGAAATTCACGGCCATCACC | 500 |
| Rep_rev1 | CCAAATGCGGTTGCCTCAAA |  |
| *race1* (reverse) | TGTGATTCCTTTCTTCTAGGTCC | ≈200 |
| *race2* (reverse) | GAGTGCACGACACCAGAGAAGG |  |

A
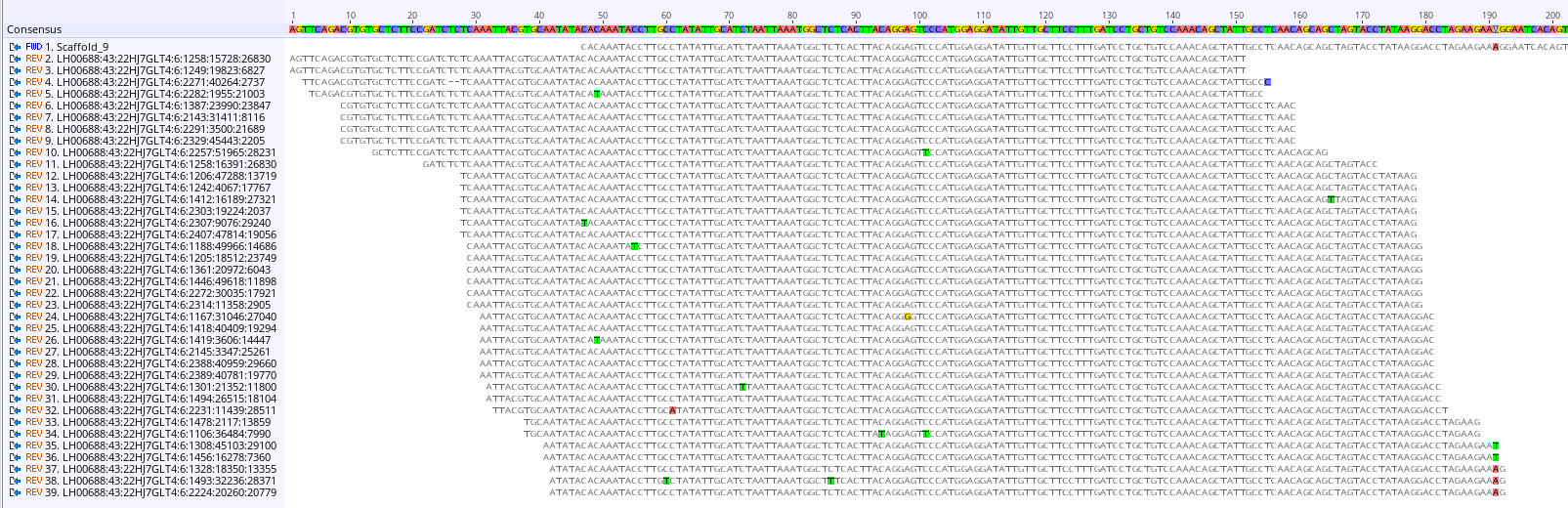


B


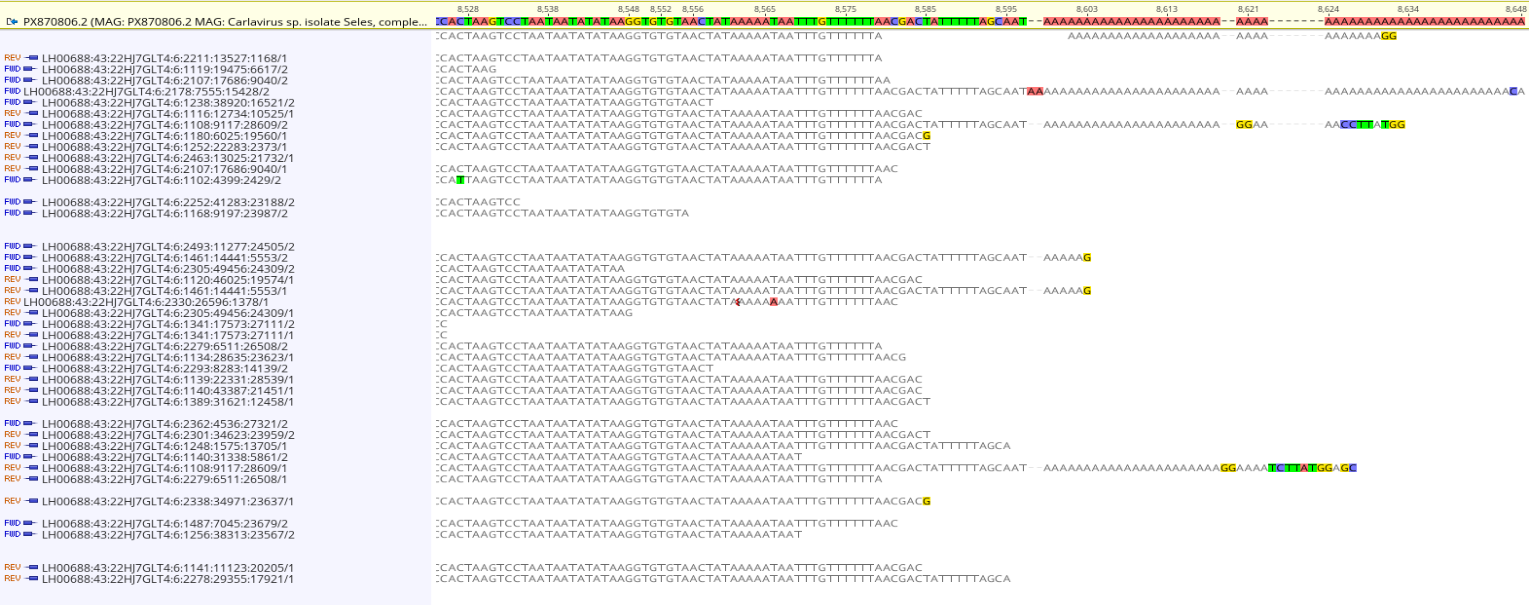


**Supplementary Figure 1.** Multiple sequence alignment (generated with Geneious Prime® 2020.0.5 (Biomatters Ltd.) showing the 5′ (A) and 3’ (B) consensus regions of RNA-seq reads mapped to the selected consensus contig of the Seles weed carlavirus. The alignment highlights the coverage and nucleotide consistency across the 5′ and 3’ untranslated regions.
